# The development of a kinematic model to quantify in-shoe foot motion

**DOI:** 10.1186/1757-1146-5-S1-O43

**Published:** 2012-04-10

**Authors:** Chris Bishop, Gunther Paul, Dominic Thewlis

**Affiliations:** 1 School of Health Sciences, University of South Australia, Adelaide, South Australia, 5000, Australia; 2 Mawson Institute, University of South Australia, Adelaide, South Australia, 5041, Australia; 3 Sansom Institute for Health Research, University of South Australia, Adelaide, South Australia, 5000, Australia

## Study aim

To develop a kinematic model to quantify in-shoe foot kinematics during gait.

## Methods and material

**Figure 1:**
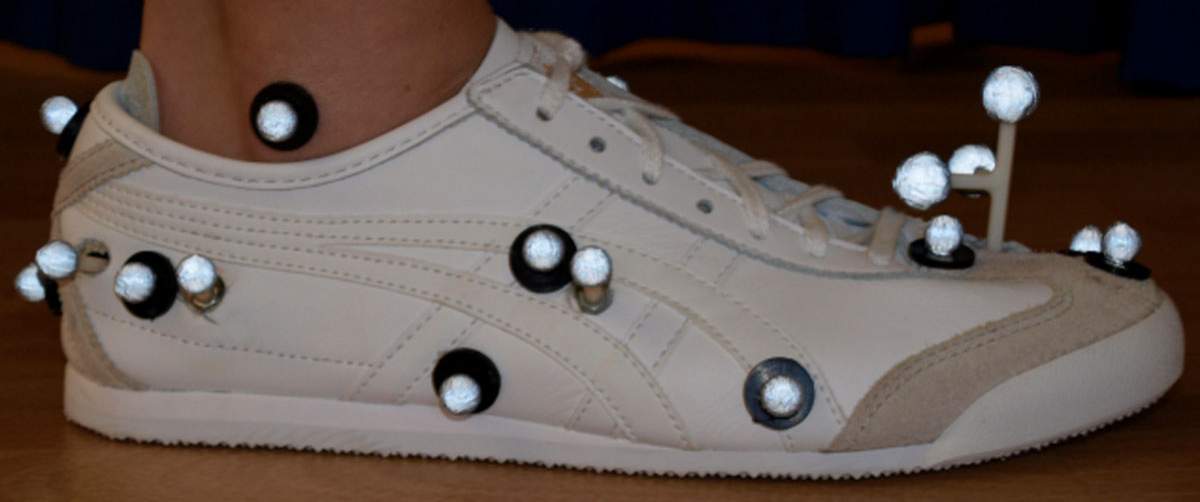
Shod and in-shoe marker set

Twenty-four participants (mean age - 21.8 yrs ± 3.5 yrs, height - 1.75 m ± 0.09 m and body mass - 71.0 kg ± 10.6 kg) were recruited. A marker set consisting of 20 x 10 mm markers was developed to track in-shoe joint kinematics [1]. Reliability and accuracy estimates of calibration marker placement on the shoe were determined. To track in-shoe foot motion, 12 mm diameter holes were punched in the shoe upper, with 25 mm marker wands mounted on the skin through the shoe (Figure 1). The marker set defined a four-segment kinematic model of the foot and ankle (shank, hindfoot, midfoot-forefoot complex and hallux). To define model parameters and moments of inertia, a CT scan was taken of 12 participant’s feet. The reconstruction of 3-D bone geometries from two-dimensional grey scale images (DICOM format) was conducted in Simpleware software. Shoe-mounted marker offsets and moments of inertia were inputted to Visual3D. The kinematics of the shoe were described before and after modification to quantify post-modification shoe integrity. The model was deemed sensitive if it detected changes in joint kinematics between conditions that were both statistically significant and greater than the calculated Standard Error of Measurement (SEM) [2].

## Results

The intra-rater (ICC = 0.68 – 0.99) and inter-rater reliability (ICC = 0.75 – 0.98) of marker placement on the shoe ranged from moderate to excellent. The error of calibration marker placement on the shoe was < 5 mm compared to skin-mounted markers.

## Conclusion

In conclusion, we present an accurate and reliable kinematic model to describe in-shoe foot kinematics during gait.
